# Dose-response association of implantable device-measured physical activity with long-term cardiac death and all-cause mortality in patients at high risk of sudden cardiac death: a cohort study

**DOI:** 10.1186/s12966-020-01026-2

**Published:** 2020-09-21

**Authors:** Xiaoyao Li, Shuang Zhao, Keping Chen, Wei Hua, Yangang Su, Jiefu Yang, Zhaoguang Liang, Wei Xu, Shu Zhang

**Affiliations:** 1 Arrhythmia Center, State Key Laboratory of Cardiovascular Disease, Fuwai Hospital, National Center for Cardiovascular Diseases, Chinese Academy of Medical Sciences and Peking Union Medical College, 167 Bei Li Shi Road, Xicheng District, Beijing, 100037 China; 2 Department of Cardiology, Shanghai Institute of Cardiovascular Diseases, Zhongshan Hospital, Fudan University, Shanghai, China; 3 Department of Cardiology, Beijing Hospital, Beijing, China; 4 Department of Cardiology, First Affiliated Hospital of Harbin Medical University, Harbin, China; 5 Department of Cardiology, Nanjing Drum Tower Hospital, Nanjing, China

**Keywords:** Physical activity, Sudden cardiac death, Dose-response association, Implantable cardioverter defibrillator, Cardiac resynchronization therapy defibrillator, Cardiac death, All-cause mortality

## Abstract

**Background:**

Cardiovascular implantable electronic devices (CIEDs) with physical activity (PA) recording function can continuously and automatically collect patients’ long-term PA data. The dose-response association of implantable cardioverter-defibrillator (ICD) and cardiac resynchronization therapy defibrillator (CRTD)-measured PA with cardiovascular outcomes in patients at high risk of sudden cardiac death (SCD) was investigated.

**Methods:**

In total, 822 patients fulfilling the inclusion criteria were included and divided into three groups according to baseline PA tertiles: tertile 1 (< 8.04%, *n* = 274), tertile 2 (8.04–13.24%, n = 274), and tertile 3 (> 13.24%, n = 274). The primary endpoint was cardiac death, the secondary endpoint was all-cause mortality.

**Results:**

During a mean follow-up of 59.7 ± 22.4 months, cardiac death (18.6% vs 8.8% vs 5.5%, tertiles 1–3, *P* < 0.001) and all-cause mortality (39.4% vs 20.4% vs 9.9%, tertiles 1–3, *P* < 0.001) events decreased according to PA tertiles. Compared with patients younger than 60 years old, older patients had a lower average PA level (9.6% vs 12.8%, *P* < 0.001) but higher rates of cardiac death (13.2% vs 8.1%, *P* = 0.024) and all-cause mortality (28.4% vs 16.7%, *P* < 0.001) events. Adjusted multivariate Cox regression analyses showed that a higher tertile of PA was associated with a lower risk of cardiac death (hazard ratio (HR) 0.41, 95% confidence interval (CI): 0.25–0.68, tertile 2 vs tertile 1; HR 0.28, 95% CI: 0.15–0.51, tertile 3 vs tertile 1, *P*_trend_ < 0.001). Similar results were observed for all-cause mortality. The dose-response curve showed an inverse non-linear pattern, and a significant reduction in endpoint risk was observed at the low-moderate PA level. The HR for cardiac death was reduced by half with 12.32% PA (177 min), and the HR for all-cause mortality was reduced by half with 11.92% PA (172 min). Subgroup analysis results indicated that older adults could benefit from PA and the range for achieving optimal benefits might be lower.

**Conclusions:**

PA monitoring may aid in long-term management of patients at high risk of SCD. More PA will generate better survival benefits, but even low-moderate PA is already good especially for older adults, which is relatively easy to achieve.

## Introduction

Sudden cardiac death (SCD) is a serious public health problem worldwide, accounting for approximately 50% of all cardiovascular deaths [1]. An implantable cardioverter defibrillator (ICD) can effectively terminate malignant tachyarrhythmia, prevent SCD and reduce all-cause mortality [2]. A number of studies have indicated that physical inactivity is a risk factor for a variety of chronic diseases [3–5], including cardiovascular morbidity and mortality [6–10].

Previous studies focusing on physical activity (PA) have mostly used self-assessment questionnaires with certain biases and errors, such as recall biases, especially for older participants, due to their education level and cognitive function [11, 12]. As smart wearable devices emerged, researchers began to use objective device-measured PA in clinical studies. However, most studies had smaller sample sizes, and the duration of continuous monitoring could only be performed for a short duration [13]. The number of patients with cardiovascular implantable electronic devices (CIEDs) is increasing noticeably. CIEDs with PA recording function can continuously and automatically collect patients’ long-term PA data. Home monitoring (HM) can detect 24-h PA, and the data are detailed and accurate with high sustainability. More recently, studies have focused on the dose-response relationship of PA and outcomes to determine the best benefit interval. The dose-response association of implantable device measured PA with cardiovascular outcomes in patients with ICDs remains unclear.

Population aging is a common global problem. The population aged 60 years or older reached 962 million in 2017, which was more than double the size of this population compared with that in 1980 [14]. Cardiac diseases are becoming the leading contributors to the disease burden in people aged 60 years and older, accounting for 30.3% of the total [15]. Older people are more likely to have exercise restrictions with decreased PA and a lower rate of compliance with guideline recommendations [16, 17]. Whether older adults with ICDs could benefit from PA and the range for achieving optimal benefits is not well known.

The present study aimed to investigate the dose-response association of ICD/cardiac resynchronization therapy defibrillators (CRTD)-measured PA with cardiovascular outcomes by long-term continuous HM and further perform subgroup analysis in younger and older adults.

## Methods

### Study population

Patients from the SUMMIT registry study (Study of Home Monitoring System Safety and Efficacy in Cardiac Implantable Electronic Device-implanted Patients) in China were retrospectively analyzed.

Patients who underwent ICD or CRTD implantation and met the inclusion criteria between May 2010 and April 2014 were included in this study. This study included patients who [1] were older than or equal to 18 years of age [2]; were eligible for an ICD/CRTD in accordance with indications specified by guidelines. These included primary prevention patients who received ICDs or CRTDs on a prophylactic basis without a prior history of SCD, cardiac arrest, or sustained ventricular tachycardia (VT) and secondary prevention patients who experienced resuscitated SCD, cardiac arrest, or sustained VT before ICD implantation; and [3] were implanted with an ICD/ CRTD (Biotronik, Germany) device with HM; and who had [4] survived more than three months after CIED implantation. The exclusion criteria were patients: [1] who were unable to follow up as required or had missing HM data [2]; with a diagnosed malignant tumor or life expectancy less than 1 year; and [3] who were scheduled for heart transplant. All equipment was programmed to provide continuous patient monitoring data. The present study complied with the Declaration of Helsinki and approved by ethics committee of Fuwai Hospital (the chief institute) and all other participating organizations, and all patients provided written informed consent before entering this study.

### PA recording

PA was measured with an integrated circuit accelerometer embedded in the pulse generator of the ICD/CRTD [18]. The time during which the motion sensors of the Biotronik devices delivered rates higher than the devices’ basic rates was recorded. The accuracy of PA measurement has been validated with treadmill test [19]. The PA resolution was 2 s, and the data were converted into % per 24 h. For example, 10% PA indicated 2.4 h of daily PA. The Biotronik remote monitoring system can automatically transmit data stored in implantable devices to the Biotronik service center every day. As the PA level early after discharge was expected to be less than usual, the data were collected during the first 30–60 days after ICDs/CRTDs implantation, in accordance with previous studies [20, 21], and the mean value of 30-day PA data was calculated as the baseline PA for each patient.

### Data collection

Baseline data for all admitted patients in this study were derived from medical records during hospitalization, and included age, gender, body mass index (BMI), New York Heart Association (NYHA) class, ICD or CRTD implantation, primary or secondary prevention indication, comorbidities (ischemic cardiomyopathy, hypertension, diabetes, stroke, atrial fibrillation (AF), vascular disease, prior myocardial infarction, and pre-implant syncope), and medication (renin-angiotensin system blockers, β receptor blockers, aldosterone antagonists, statins, loop-diuretics, digoxins, and amiodarone). Echocardiography parameters such as left ventricular ejection fraction (LVEF) and left ventricular end-diastolic diameter (LVEDD) were evaluated by two experienced echocardiography physicians. And LVEF was calculated using the modified Simpson’s biplane rule.

### Groups

All enrolled patients were divided into three groups according to baseline PA level tertiles: tertile 1 (< 8.04%, *n* = 274), tertile 2 (8.04–13.24%, n = 274), and tertile 3 (> 13.24%, n = 274). According to the guideline for age classification in China and a previous study, patients aged 60 years or older were defined as the older group [22].

### Endpoints

Regular follow-up was conducted with all enrolled patients. If the patient’s daily transmission was interrupted, the clinical research coordinator immediately confirmed the patient’s status by contacting the family. The cause of death was based on the death certificate. The primary endpoint of the present study was cardiac death (*ICD-10* I00 to I09, I11, I20 to I51), and the secondary endpoint was all-cause mortality.

### Statistical methods

Continuous variables are presented as means±SDs, and categorical variables are presented as numbers and percentages. Baseline characteristics were compared among the groups using one-way analysis of variance for continuous variables and the Chi-square test for categorical variables. Cardiac death and all-cause mortality were calculated, and the difference was compared between groups with a Chi-square test. Cox proportional hazard regression analysis was used to evaluate the association between different PA groups for endpoint events. Hazard ratios (HRs) and 95% confidence intervals (CIs) were calculated to show the impact. Associations were investigated with stratification according to baseline age. Model 1 was adjusted for age and gender. Model 2 was further adjusted for primary prevention, NYHA class, CRTD implantation, LVEF, LVEDD, β-blockers, and aldosterone antagonists. Model 3 was adjusted for factors in Model 2 and potential mediators on the causal pathway including BMI, ischemic cardiomyopathy, hypertension, AF, diabetes, and prior myocardial infarction. In addition, a restricted cubic spline was used to assess the dose-response association between PA and the risk of endpoints. Four knots were placed at the 5th, 35th, 65th, and 95th percentiles of PA. To specify the PA range for achieving optimal benefits as a target value that can be practicable in clinical practice, we determined the amount of PA required when the risk was halved, and 8.04% PA (lower tertile point) was used as the reference (HR = 1.0). A value of *P* < 0.05 was considered significant in all conditions. Statistical analyses were performed using SAS v.9.4 (SAS Institute, Cary, NC, USA) and STATA v12.0 (STATA Corp., College Station, TX, USA).

## Results

### Baseline characteristics

Among a total of 1008 patients, 845 patients with PA data were obtained. Nineteen patients with incomplete data, 1 patient lost to follow-up, and 3 patients who died within 3 months after implantation were excluded. A total of 822 patients fulfilling the admission criteria were finally analyzed.

Men were dominant in the study cohort (73.8%). The average age was 60.8 ± 13.8 years, and the mean baseline PA level was 11.0 ± 5.8% (range 0.02–37.66%). The cohort was divided into three groups according to baseline PA tertiles. Table 1 illustrates the baseline characteristics of the participants.

**Table 1:** Baseline Clinical Characteristics

	Total (***n*** = 822)	Tertile 1 (***n*** = 274)	Tertile 2 (n = 274)	Tertile 3 (n = 274)	***P*** value
**Demographics**					
Male	607 (73.8)	187 (68.3)	206 (75.2)	214 (78.1)	0.026
Physical activity, %	11.0 ± 5.8	4.9 ± 2.0	10.6 ± 1.5	17.5 ± 3.9	< 0.001
Age at implantation, years	60.8 ± 13.8	65.5 ± 13.2	61.1 ± 13.4	55.7 ± 13.1	< 0.001
BMI, kg/m2	23.6 ± 3.4	23.5 ± 3.7	23.5 ± 3.5	23.8 ± 3.4	0.293
Primary prevention	434 (52.8)	146 (53.3)	146 (53.3)	142 (51.8)	0.925
NYHA, class I–II	420 (51.1)	106 (38.7)	143 (52.2)	171 (62.4)	< 0.001
CRTD	217 (26.4)	82 (29.9)	76 (27.7)	59 (21.5)	0.069
**Comorbidities**					
Ischemic cardiomyopathy	281 (34.2)	115 (42.0)	103 (37.6)	63 (23.0)	< 0.001
Hypertension	259 (31.5)	92 (33.6)	96 (35.0)	71 (25.9)	0.047
Diabetes	78 (9.5)	39 (14.2)	20 (7.3)	19 (6.9)	0.005
Stroke	16 (1.9)	10 (3.7)	3 (1.1)	3 (1.1)	0.044
Atrial fibrillation	90 (10.9)	33 (12.0)	31 (11.3)	26 (9.5)	0.615
Valvular disease	20 (2.4)	9 (3.28)	8 (2.92)	3 (1.09)	0.204
Prior myocardial infarction	128 (15.6)	60 (21.9)	45 (16.4)	23 (8.4)	< 0.001
Pre-implant syncope	175 (21.3)	57 (20.8)	52 (19.0)	66 (24.1)	0.334
**Echocardiography**					
LVEF, %	42.7 ± 14.9	40.2 ± 14.6	42.9 ± 14.5	44.9 ± 15.3	< 0.001
LVEDD, mm	58.6 ± 13.1	58.9 ± 12.0	58.7 ± 13.6	58.1 ± 13.6	0.586
**Medication**					
Beta-blockers	507 (61.7)	169 (61.7)	167 (61.0)	171 (62.4)	0.940
ACEIs/ARBs	321 (39.1)	116 (42.3)	106 (38.7)	99 (36.1)	0.327
Aldosterone antagonists	295 (35.9)	14 (45.3)	96 (35.0)	75 (27.4)	< 0.001
Statins	192 (23.4)	71 (25.9)	65 (23.7)	56 (2.4)	0.313
Loop diuretics	340 (41.4)	134 (48.9)	119 (43.4)	87 (31.8)	< 0.001
Digoxins	170 (20.7)	68 (24.8)	54 (19.7)	48 (17.5)	0.096
Amiodarone	250 (30.4)	87 (31.8)	84 (30.7)	79 (28.8)	0.755

*Abbreviations*: *ACEIs* angiotensin-converting enzyme inhibitors, *ARBs* angiotensin receptor blockers, *BMI* Body Mass Index, *CRTD* cardiac resynchronization therapy and implantable cardioverter-defibrillator, *LVEDD* left ventricular end-diastolic dimension, *LVEF* left ventricular ejection fraction, *NYHA class* New York Heart Association class

Significant differences among the three groups were detected for male gender (*P* = 0.026), age at implantation (*P* < 0.001), NYHA class (*P* < 0.001), LVEF (*P* < 0.001), ischemic cardiomyopathy (*P* < 0.001), hypertension (*P* = 0.047), diabetes (*P* = 0.005), stroke (*P* = 0.044), prior myocardial infarction (*P* < 0.001), and use of aldosterone antagonists (*P* < 0.001) and loop-diuretics (*P* < 0.001). No significant differences were found regarding other baseline characteristics (Table 1).

### Clinical outcomes

The mean follow-up time was 59.7 ± 22.4 months. A total of 90 cardiac deaths (10.9%) and 191 all-cause mortality events (23.2%) occurred. The percentage of cardiac death (18.6% vs 8.8% vs 5.5%, tertiles 1–3, *P* < 0.001) and all-cause mortality (39.4% vs 20.4% vs 9.9%, tertiles 1–3, *P* < 0.001) events decreased according to baseline PA tertiles.

**Fig. 1:**
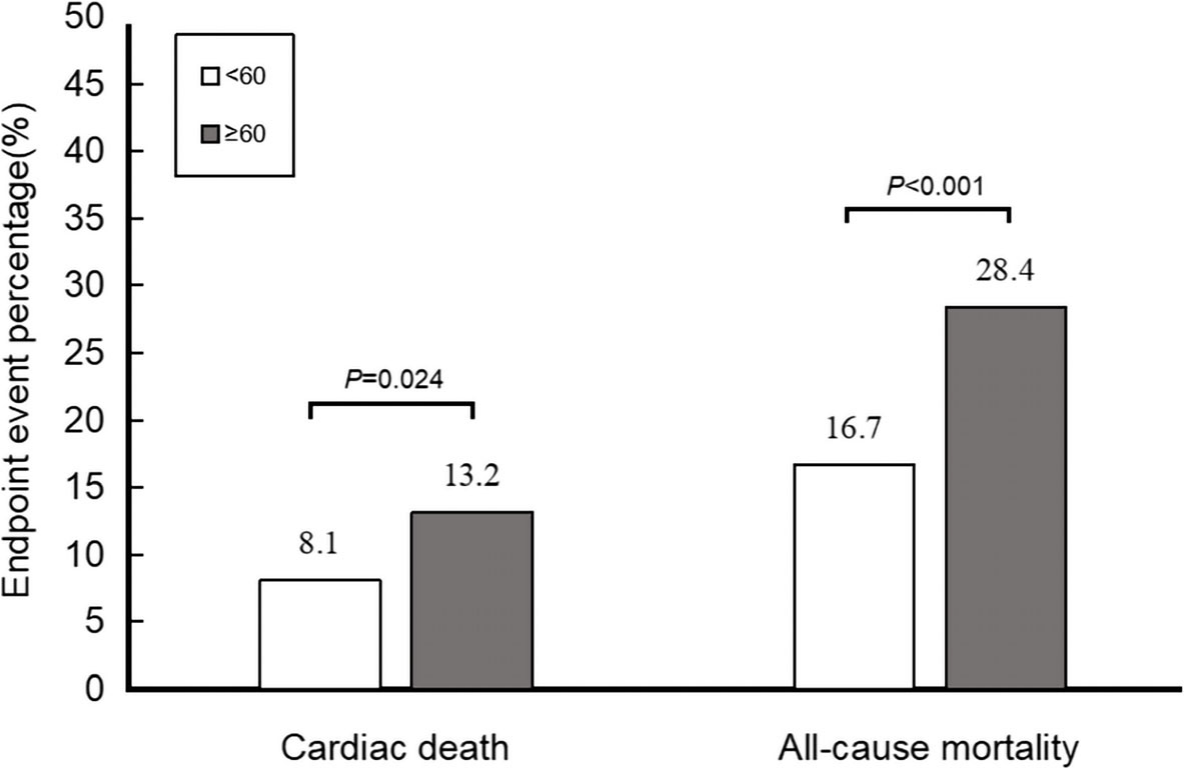
Cardiac death and all-cause mortality events percentage in younger and older groups

A total of 462 patients were aged 60 years or older (56.2%). Compared to patients younger than 60 years, older patients had a lower average PA level (9.6% vs 12.8%, *P* < 0.001) but higher rates of cardiac death (13.2% vs 8.1%, *P* = 0.024) and all-cause mortality (28.4% vs 16.7%, *P* < 0.001) events (Fig. 1).

### PA and cardiac death

Multivariate Cox regression analyses showed that a higher level of PA was inversely associated with cardiac death (HR 0.40, 95% CI: 0.25–0.66, tertile 2 vs tertile 1; HR 0.25, 95% CI: 0.14–0.45, tertile 3 vs tertile 1; *P*_trend_ < 0.001). The results remained statistically significant after adjustment for confounders including age, gender, primary prevention, NYHA class, CRTD implantation, LVEF, LVEDD, β-blocker use, and aldosterone antagonist use (Model 2). After additional adjustment of potential mediators, including BMI, ischemic cardiomyopathy, hypertension, AF, diabetes, and prior myocardial infarction, the results were similar (Model 3). The results from Model 2 and Model 3 were consistent, and as obesity and comorbidities are very common and related to clinical diagnosis and treatment decisions for ICD patients, in the present study, findings from Model 3 were used as the main results (Table 2).

**Table 2:** Cardiac death outcomes and multivariate cox regression analyses

	No. of events	No. of participants	Model 1	Model 2	Model 3	*P*_trend_
Tertile 1	51	274	Ref.	Ref.	Ref.	< 0.001
Tertile 2	24	274	0.40 (0.25–0.66)	0.42 (0.26–0.69)	0.41 (0.25–0.68)	
Tertile 3	15	274	0.25 (0.14–0.45)	0.26 (0.14–0.48)	0.28 (0.15–0.51)	
Age, years						
< 60						
Tertile 1	10	76	Ref.	Ref.	Ref.	0.127
Tertile 2	11	121	0.57 (0.24–1.35)	0.76 (0.31–1.85)	0.82 (0.33–2.04)	
Tertile 3	8	163	0.29 (0.11–0.74)	0.39 (0.15–1.06)	0.47 (0.17–1.26)	
≥ 60						
Tertile 1	41	198	Ref.	Ref.	Ref.	< 0.001
Tertile 2	13	153	0.35 (0.19–0.65)	0.34 (0.18–0.65)	0.34 (0.18–0.64)	
Tertile 3	7	111	0.25 (0.11–0.57)	0.24 (0.11–0.55)	0.25 (0.11–0.57)	

Model 1 adjusted for age and gender; Model 2 further adjusted for Model 1 puls primary prevention, NYHA, CRT-D, LVEF, LVEDD, β-blockers, and aldosterone antagonists; Model 3 adjusted factors in Model 2 and potential mediators on the causal pathway including BMI, ischemic cardiomyopathy, hypertension, AF, diabetes, prior myocardial infarction

### PA and all-cause mortality

The dose-response association of PA with all-cause mortality was similar, as shown in Table 3. A higher PA level was significantly related to a lower risk of all-cause mortality (HR 0.46, 95% CI: 0.33–0.64, tertile 2 vs tertile 1; HR 0.23, 95% CI: 0.15–0.35, tertile 3 vs tertile 1) in a dose-response pattern (Model 1, *P*_trend_ < 0.001). In Model 2 and Model 3, the associations between PA and all-cause mortality were similar, and results from Model 3 were used as the main results (Table 3).

**Table 3:** All-cause mortality outcomes and multivariate cox regression analyses

	No. of events	No. of participants	Model 1	Model 2	Model 3	*P*_trend_
Tertile 1	108	274	Ref.	Ref.	Ref.	< 0.001
Tertile 2	56	274	0.46 (0.33–0.64)	0.47 (0.34–0.66)	0.46 (0.33–0.64)	
Tertile 3	27	274	0.23 (0.15–0.35)	0.24 (0.15–0.37)	0.24 (0.16–0.38)	
Age, years						
< 60						
Tertile 1	26	76	Ref.	Ref.	Ref.	< 0.001
Tertile 2	20	121	0.40 (0.22–0.72)	0.55 (0.30–1.01)	0.55 (0.30–1.01)	
Tertile 3	14	163	0.21 (0.11–0.40)	0.28 (0.14–0.56)	0.29 (0.15–0.59)	
≥ 60						
Tertile 1	82	198	Ref.	Ref.	Ref.	< 0.001
Tertile 2	36	153	0.49 (0.33–0.73)	0.47 (0.31–0.70)	0.46 (0.31–0.70)	
Tertile 3	13	111	0.24 (0.13–0.44)	0.23 (0.12–0.41)	0.23 (0.13–0.43)	

Model 1 adjusted for age and gender; Model 2 further adjusted for Model 1 puls primary prevention, NYHA, CRT-D, LVEF, LVEDD, β-blockers, and aldosterone antagonists; Model 3 adjusted factors in Model 2 and potential mediators on the causal pathway including BMI, ischemic cardiomyopathy, hypertension, AF, diabetes, prior myocardial infarction

### PA range for achieving optimal benefits regarding cardiac death and subgroup analysis of younger and older adults

**Fig. 2:**
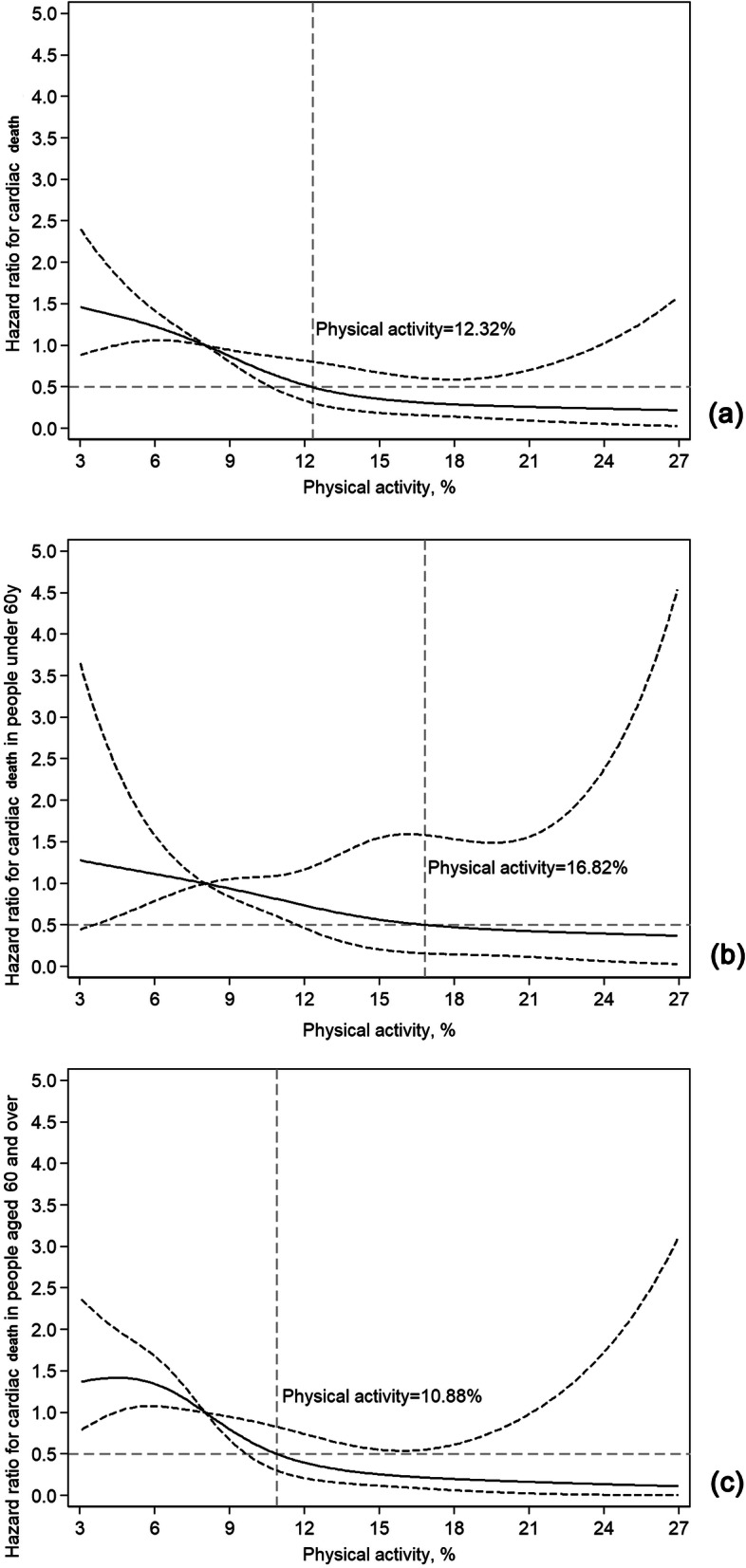
Dose-response curve of PA and cardiac death in total and different age groups; PA, physical activity. The bold and the dashed lines represent the estimated risk ratio (hazard ratio, HR) and the 95% confidence interval, respectively. The horizontal dashed line indicates that the HR value is 0.5, and the intersection of the vertical dashed line and the curve indicates the corresponding PA value

To further investigate the association of PA with the endpoints, dose-response curves were constructed, and a subgroup analysis of older and younger adults was performed. As shown in Fig. 2a, a significant reduction in cardiac death risk was observed at low-moderate PA levels. The risk was halved when the PA level was 12.32% (approximately 177 min), after which additional benefit of more PA was quite limited (Fig. 2a). Subgroup analysis showed that older patients could also benefit from PA, and low-moderate PA reduced the risk of cardiac death in older adults more rapidly than in younger adults. For example, using the same amount of PA as a reference, younger patients needed 16.82% PA (approximately 242 min) to achieve half of the risk, while older patients only need 10.88% PA (approximately 157 min) (Fig. 2 b and c).

### PA range for achieving optimal benefits for all-cause mortality and subgroup analysis of younger and older adults

**Fig. 3:**
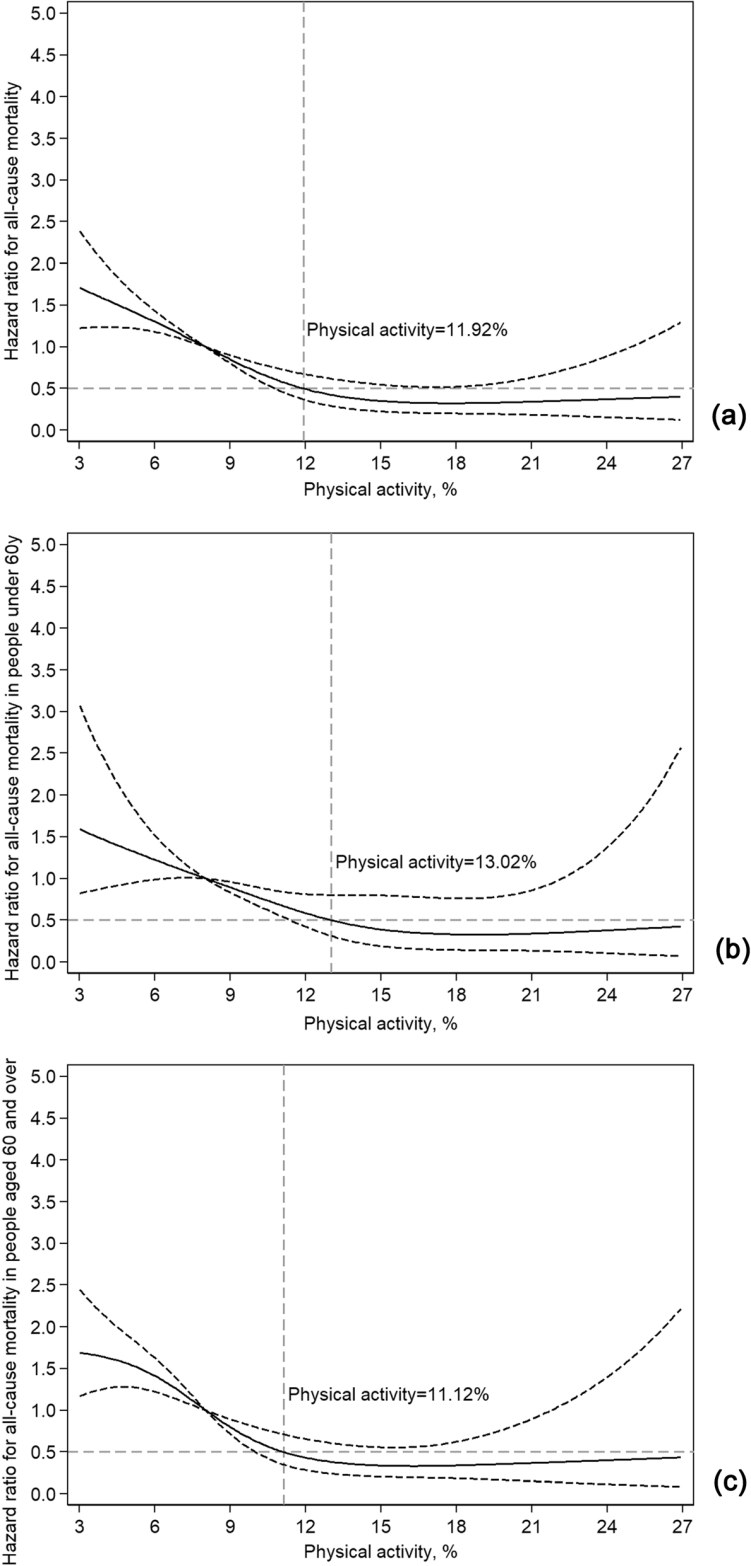
Dose-response curve of PA and all-cause mortality in total and different age groups; PA, physical activity. The bold and the dashed lines represent the estimated risk ratio (hazard ratio, HR) and the 95% confidence interval, respectively. The horizontal dashed line indicates that the HR value is 0.5, and the intersection of the vertical dashed line and the curve indicates the corresponding PA value

The association of PA and all-cause mortality was similar, as shown in Fig. 3. A significant reduction in all-cause mortality risk was observed at the low-moderate level of PA (the HR was halved with 11.92% PA, approximately 172 min). Similarly, subgroup analysis showed that this dose-response association remained in older patients. To obtain half of the risk of all-cause mortality, younger patients needed 13.02% PA (approximately 187 min), while older patients only need 11.12% PA (approximately 160 min) (Fig. 3b and c).

## Discussion

The main findings of the present study were as follows. First, there was an inverse dose-response association of ICD/CRTD-measured PA with long-term cardiac death and all-cause mortality in patients at high risk of SCD. Second, the association exhibited a non-linear pattern, and a significant reduction in cardiac death and all-cause mortality risk was observed with a low-moderate level of PA. Third, subgroup analysis results indicated that older adults could benefit from PA, and the range for achieving optimal benefits might be lower.

We demonstrated the dose-response association of PA with cardiovascular outcomes. This finding was consistent with previous studies. Schnohr et al. found an inverse dose-response relationship between PA and both coronary heart disease and all-cause mortality in healthy individuals [23]. Joseph et al. observed PA had an inverse dose-response effect on all-cause mortality in hypertension patients [24]. In addition, Ekelund published a meta-analysis confirming the dose-response association between wearable accelerometry measured PA and all-cause mortality [13]. However, in those studies, PA was based on questionnaires with low accuracy or a wearable device with a short detection period. In contrast, our study was conducted in patients at high risk of SCD risk with ICDs/CRTDs that had continuous PA recording function [19, 25]. HM technology allowed the instantaneous transmission of stored device data and enabled the continuous and longer acquisition of PA data. In addition, the present study conducted a long-term follow-up of the target population, and the real-time status of each patient could be obtained through remote HM.

Previous studies focusing on implantable device-measured PA in ICDs/CRTDs patients did not describe its dose-response association with cardiovascular outcomes. Kramer et al. found an increase in baseline PA was associated with reduced all-cause mortality in patients with ICDs [21]. Zhao et al. verified the relationship of PA with cardiac death and provided a cut-off value [20]. Based on those previous studies, we further found an inverse non-linear dose-response association in patients ICDs/CRTDs and a significant reduction in cardiac death/all-cause mortality risk was observed with low-moderate PA levels. This finding was consistent with most previous studies regarding the dose-response pattern of PA [6, 7, 13]. However, Cheng et al. found a linear dose-response association and high PA had more obvious cardiovascular benefits than moderate PA [26]. Hupin et al. concluded the greatest reduction in risk occurred in those who changed from performing no moderate-to-vigorous physical activity (MVPA) to performing some MVPA [27]. The level of PA and the benefit pattern might depend on the person’s health status and ability to perform PA. The population examined in the present study had severe heart disease, and the amount of total PA might be lower than those participants in the studies mentioned above.

According to current guidelines, the recommended PA amount for older adults duplicate those for younger adults [28]. However, for many older adults, the recommended amount of PA may be excessive, explaining why the compliance rate of older individuals, is extremely low [16, 17, 29]. Researches on the benefit of PA in the older population were inconsistent. Cheng et al. concluded the benefit of PA was decreased for those aged over 65 years [26]. Another study showed older adults needed higher moderate and high-intensity exercise to gain benefit [30]. Hupin suggested even low doses of MVPA should be encouraged for older adults in their daily lives. The present study found older patients with ICDs/CRTDs could obviously benefit from PA and a significant reduction in cardiac death and all-cause mortality risk was observed with low-moderate level of PA. Older patients might need less dose of PA to reduce the risk of all-cause mortality and cardiac death by half. Therefore, for older adults, especially those who are at risk of sudden death and implanted with ICD, PA is worth recommending, and attention should be paid to the PA range resulting in optimal benefits which was easy to achieve. This result might be due to the decrease in the overall metabolic intensity of older adults.

Modern medicine has made great progress in many aspects, and patients are currently receiving improved treatment with novel drugs and devices. However, PA is a safe, inexpensive, easily accessible, and environmentally friendly therapy that patients often fail to implement. Our results demonstrated the importance of maintaining a certain level of daily PA in people already suffering from severe heart diseases. The range required for optimal benefits is not very high, especially for older adults, and is relatively easy to achieve. In clinical practice, it is important to understand the range of PA which could achieve optimal benefits. In addition, PA monitoring is very effective and can be introduced for all patients with CIEDs. PA monitoring can be further used in long-term management of patients with cardiovascular and even other chronic diseases.

### Limitations

The present study analyzed the dose-response association of objective PA and the long-term prognosis of patients at high risk of SCD. Nevertheless, several limitations should be stated. First, we only included patients with implanted devices, which might cause selection bias. Second, despite of adjustment for multiple covariates, we did not take socioeconomic status (SES) into consideration, and several adjusted variables could be potential mediators. The possibility of overadjustment bias in Model 3 should be noted. Lastly, the conclusions were based on reverse causation, and residual confounding may still exist, thus more prospective studies with larger samples are needed to further validate our findings.

## Conclusions

PA monitoring may aid in long-term management of patients at high risk of SCD. More PA will generate better survival benefits, but even low-moderate PA is already good especially for older adults, which is relatively easy to achieve.

## Data Availability

The datasets generated and analyzed during the current study are not publicly available due to the Fuwai Hospital regulations, but are available from the corresponding author on reasonable request.

## Abbreviations

ACEIs: Angiotensin-converting enzyme inhibitors
ARBs: Angiotensin receptor blockers
BMI: Body Mass Index
CI: Confidence interval
CIED: Cardiovascular implantable electronic devices
CRTD: Cardiac resynchronization therapy defibrillator
HM: Home monitoring
HR: Hazard ratio
ICD: Implantable cardioverter-defibrillator
LVEDD: Left ventricular end-diastolic dimension
LVEF: Left ventricular ejection fraction
MVPA: Moderate-to-vigorous physical activity
NYHA: New York Heart Association
PA: Physical activity
SCD: Sudden cardiac death
SES: Socioeconomic status
VT: Ventricular tachycardia
